# Knowledge, attitudes, and practices regarding dengue and its vectors among medical professionals: a cross-sectional study

**DOI:** 10.3389/fcimb.2025.1560054

**Published:** 2025-04-30

**Authors:** Rania Ali El Hadi Mohamed, Yasir Khan, Khalid J. Alzahrani, Fuad M. Alzahrani, Khalaf F. Alsharif, Aamir Khan, Fazal Noor, Abdul Qadeer, Geng-Bai Lin, Chien-Chin Chen

**Affiliations:** ^1^ Department of Biology, College of Science, Princess Nourah bint Abdulrahman University, Riyadh, Saudi Arabia; ^2^ Lady Reading Hospital, Peshawar, Khyber Pakhtunkhwa, Pakistan; ^3^ Department of Clinical Laboratories Sciences, College of Applied Medical Sciences, Taif University, Taif, Saudi Arabia; ^4^ Livestock and Dairy Development Department, Extension Wing, Peshawar, Khyber Pakhtunkhwa, Pakistan; ^5^ Livestock and Dairy Development Department, Research Wing, Peshawar, Khyber Pakhtunkhwa, Pakistan; ^6^ Department of Cell Biology, School of Life Sciences, Central South University, Changsha, China; ^7^ Division of Surgical Critical Care, Department of Surgery, Ditmanson Medical Foundation Chia-Yi Christian Hospital, Chiayi, Taiwan; ^8^ Department of Pathology, Ditmanson Medical Foundation Chia-Yi Christian Hospital, Chiayi, Taiwan; ^9^ Department of Cosmetic Science, Chia Nan University of Pharmacy and Science, Tainan, Taiwan; ^10^ Doctoral Program in Translational Medicine, National Chung Hsing University, Taichung, Taiwan; ^11^ Department of Biotechnology and Bioindustry Sciences, College of Bioscience and Biotechnology, National Cheng Kung University, Tainan, Taiwan

**Keywords:** Dengue, One Health, public health, perception, attitude

## Abstract

Dengue fever remains a significant global public health issue, necessitating a collaborative One Health strategy for efficient management. This cross-sectional study evaluated the knowledge, attitudes, and practices (KAP) of 516 healthcare professionals regarding dengue and its vectors. Most participants were young (82.6%), male (75.6%), and had MBBS qualifications (61.4%), predominantly employed in hospital settings (70.3%), and living in non-hotspot areas (53.3%). The research indicated that 65.1% of respondents possessed moderate knowledge about dengue, while 19.6% exhibited high knowledge and 15.3% showed low knowledge. Most participants (87.8%) correctly identified *Aedes* mosquitoes as the main vector, with 52.7% recognizing stagnant clean water as their breeding habitat. Positive attitudes were noted among 72.5% of participants; 51% viewed dengue as a moderately serious threat, and 47.3% strongly agreed on the crucial role of healthcare professionals in prevention. Conversely, 48.9% pointed out insufficient preventive measures in their facilities. In terms of practices, 80.8% of participants regularly informed patients about prevention, and 79.1% recommended CBC tests for suspected cases. Protective measures such as repellents and mosquito nets were consistently utilized by 57.2%, whereas 41.1% reported infrequent use. Remarkably, 59.7% had never undergone formal training on dengue prevention and management, with only 23.6% receiving consistent training. Furthermore, while 50.8% indicated that their facilities had adequate resources for dengue treatment, 42.8% reported a lack of mosquito control activities. These findings underline significant gaps in training, resource availability, and preventive practices, highlighting the urgent need for enhanced capacity-building, resource allocation, and intersectoral collaboration within the One Health framework to combat the effects of dengue in the region.

## 1 Introduction

Dengue infection is recognized as a significant global health concern (Bhatt et al., 2013). Over 50% of the global population resides in areas at risk for dengue transmission, highlighting its significant role in global morbidity and mortality (Obi et al., 2021). Dengue is a viral infection transmitted by mosquitoes, caused by one of four distinct serotypes of the dengue virus (DENV). It primarily occurs in tropical and subtropical regions where its main vector, *Aedes aegypti*, flourishes. Each year, approximately 100 million symptomatic cases are reported (Bhatt et al., 2013).

With climate change and urbanization, the likelihood of dengue transmission is expected to rise dramatically over the next fifty years, affecting nearly half of the global population residing in regions favorable to dengue spread (Messina et al., 2019). The World Health Organization (WHO) has classified dengue and dengue hemorrhagic fever (DHF) as endemic to the Asian subcontinent, with the disease now affecting 112 countries globally (Malhotra and Kaur, 2014).

A range of vector control methods has been employed to lower the *Aedes* mosquito population. These methods include fogging, introducing fish that eat mosquito larvae, eliminating breeding sites, applying larvicides, releasing genetically modified *Aedes* mosquitoes to reduce reproduction, and infecting female *Aedes* mosquitoes with *Wolbachia*. Nonetheless, the success of these strategies has been restricted (Frentiu et al., 2014). Dengvaxia (CYD-TDV), the sole approved dengue vaccine, has notable drawbacks stemming from its insufficient effectiveness against the DENV-1 and DENV-2 serotypes, alongside a potential risk of severe dengue in those who have yet to be exposed to the virus (Hadinegoro et al., 2015).

Dengue fever epidemic affected much of Asia and the Pacific during the 20th century (Yang et al., 2021). The dengue virus first entered Asia via South Asian nations (Agampodi and Wickramage, 2013). The initial recorded outbreak of DHF in Asia took place in the 1950s in the Philippines and Thailand. In the following twenty years, the disease proliferated across Southeast Asia, resulting in DHF becoming the primary cause of pediatric hospitalizations and deaths by the mid-1970s. In the 1980s and 1990s, dengue transmission escalated, with epidemics occurring every 3 to 5 years in hyperendemic regions (Thi, 2015).

In Pakistan, the first confirmed dengue outbreak, attributed to serotype DV-2, was reported in 1994 by Aga Khan University Hospital (AKUH) (Khan and Khan, 2015). Subsequently, sporadic cases of dengue hemorrhagic fever (DHF) were reported across different regions of the country. In particular, serotypes DV-1 and DV-2 were identified in the blood samples of children exhibiting undifferentiated fever (Sreerama, 2015). The swift geographic expansion and versatility of *Aedes aegypti* and *Aedes albopictus* have greatly fueled the concerning rise in dengue cases in Pakistan (Khan et al., 2018). Dengue outbreaks in the country follow a cyclical pattern, happening every 2 to 3 years, and have shown an eightfold increase in reported cases over the last decade. For example, Peshawar, Khyber Pakhtunkhwa’s (KP) third most populous city, faced its initial severe dengue epidemic in 2017, with 24,938 cases and 70 deaths recorded (Ali Mohamud et al., 2020). In 2021, another outbreak occurred, leading to more than 10,000 cases and 10 fatalities (Khan et al., 2022). The inaugural recorded dengue fever outbreak in Khyber Pakhtunkhwa (KP) took place in Swat in August 2013. During that year, KP registered the highest number of dengue infections nationwide, totaling 3,177 cases (Qamash et al., 2021).

Several social factors, such as education levels, household conditions, overcrowding, sanitation and water storage practices, electricity access, vegetation density, human behaviors, and the presence of Aedes mosquitoes influence dengue transmission worldwide (Gurevitz et al., 2021). Successful disease control depends on equipping communities with adequate information about dengue prevention methods, given that human behavior dramatically influences the conditions that favor vector breeding, blood-feeding, and disease spread (Selvarajoo et al., 2020). Communities with higher socioeconomic status (SES) generally exhibit a greater awareness of dengue, encompassing knowledge, attitudes, and preventive measures. Studies show that these communities have more success in managing the disease (Selvarajoo et al., 2020). In Selangor, Malaysia, human behavior plays a key role in the spread and transmission of dengue (Ghani et al., 2019).

In Pakistan, the public health response to dengue outbreaks mainly depends on emergency actions like widespread insecticide spraying (Rahman et al., 2021). In dengue-prone areas, vector surveillance programs have been implemented under the direction of the National Institute of Health (NIH) in Islamabad. These include ovitraps, larval and pupal sampling, as well as adult mosquito traps (Khan et al., 2015). These initiatives seek to issue early alerts for dengue and other diseases transmitted by mosquitoes (Aliya et al., 2019). Integrated vector management (IVM) strategies have recently been adopted, emphasizing habitat modification, appropriate waste disposal practices like tire removal, and boosting community awareness (Mukhtar et al., 2018).

Earlier studies have mainly concentrated on the epidemiology of dengue in the Peshawar district (Ali et al., 2019). This study marks the inaugural effort to assess the knowledge, attitudes, and practices (KAP) about dengue among healthcare professionals in Khyber Pakhtunkhwa (KP) province. In developing nations like Pakistan, preventable illnesses such as dengue pose a serious challenge to public health, often leading to elevated mortality rates. Despite the gravity of the situation, there is a scarcity of evidence regarding adults’ awareness and preventive practices related to dengue fever. To fill this void, our research aimed to evaluate healthcare professionals’ knowledge, attitudes, and perceptions in KP concerning dengue transmission, symptoms, and prevention. In addition, we aimed to assess their awareness of the necessary preventive measures to curb the spread of dengue fever in the area.

## 2 Materials and methods

### 2.1 Study design

We created an online questionnaire to gather data for this cross-sectional study. Targeting Pakistan’s medical and veterinary professions, we conducted a web-based survey from September 1 to September 30, 2024. To calculate the needed sample size, we used a response distribution of 5% and a 95% margin of error. Consequently, we aimed for about 385 respondents; however, for more precise results, we ultimately collected 516 survey responses.

### 2.2 Data collection tool

An online survey was conducted to gather the necessary data. We employed Cronbach’s Alpha test to assess the reliability of the knowledge regarding Dengue. Questions were independently formulated to evaluate the related risk factors based on knowledge, attitudes, and perceptions about the disease. The Cronbach’s Alpha scores were 0.82 for knowledge, 0.76 for attitudes, and 0.72 for perceptions, indicating that the questions assessing these areas exhibit strong internal consistency. A random sampling method was utilized for participant selection, ensuring only one email address per respondent. Participants received instructions to complete the questionnaire on either a computer or mobile device. The questionnaire was organized into four sections. The first section gathered data on age, gender, qualifications, years of experience, professional working setting, and zone of duty. The second section included ten multiple-choice questions aimed at measuring knowledge. The third section contained six questions to assess attitudes, while the fourth section included nine questions evaluating participants’ perceptions (practices) regarding the link between dengue and risk factors. Respondents were provided with response options of “Yes,” “No,” “Not sure,” and multiple-choice for each question. The survey was conducted in English.

### 2.3 Data collection procedure

The survey did not collect personal details such as names, addresses, or other identifying information about the respondents. A specific link to a structured questionnaire was created and distributed through social media. It was shared within various groups on these platforms, with requests made to both admins and group members to help circulate the link to gather sufficient responses. Before participating, each respondent had to confirm their informed consent by acknowledging a provided declaration. This consent statement stated: “After reviewing the study’s objectives, I voluntarily agree to take part in this survey and provide my answers willingly and thoughtfully.” Respondents completed the survey by clicking the “submit” button on the platform, allowing for effective data collection. To guarantee the reliability and integrity of the data, all survey questions were made mandatory.

### 2.4 Study variables

The assessment consisted of 25 questions, each offering multiple response options, aimed at evaluating knowledge about dengue, along with attitudes and perceptions regarding related risk factors. Ten questions specifically measured knowledge of dengue and its vector, with each correct answer earning 1 mark and incorrect responses receiving 0. Respondents scoring 5 or less were classified as having low knowledge, scores of 6–7 indicated moderate knowledge, and scores of 9–10 suggested high knowledge levels. To gauge respondents’ attitudes, six questions were included in the questionnaire, where each correct answer earned 3 marks, and incorrect answers received 0. The total score from these questions added up to 18 and was categorized as follows: (1) 0–6 indicates a negative attitude, (2) 7–12 signifies a moderate attitude, and (3) 13–18 suggests a positive attitude. Lastly, to evaluate practices related to dengue management and vector control, nine questions were incorporated into the survey, where each question was worth 3 marks. The scores were categorized into three groups: scores of 15 or below indicated low perception, scores between 16 and 21 reflected moderate perception, and scores of 22 or higher indicated high perception.

The age groups were defined as follows: 25 to 35 years, 36 to 45 years, 46 to 55 years, and 56 years or older. Gender was classified as either male or female. Education levels were categorized into five types: Bachelor (MBBS), Bachelor (BDS), Master, Doctorate, and Post-Doctorate. Experience was evaluated annually and divided into four ranges: less than 5 years, 5–10 years, 11–15 years, and 15 years or more. Professions were grouped into three categories: Physician, Public Health Officer, and Other. Two additional variables were considered to determine the participants’ workplace settings (Hospital, Clinic, Community Health Center, and Others) and the duty zone as either hot or non-hot spot.

### 2.5 Statistical analysis

The data gathered through Google Forms was exported into Microsoft Excel for analysis. Statistical evaluations were performed using SPSS version 2023. Variables were coded within SPSS, and Missing Value Analysis was utilized to identify any missing data. Based on the key objective of the cross-sectional study- evaluating knowledge, attitudes, and practices (KAP) of 516 healthcare professionals regarding dengue and its vectors, we adopted descriptive statistics that include frequency and percentage to illustrate participants’ socio-demographic characteristics and their knowledge, attitudes, and perceptions (KAP) regarding dengue. Association between demographic variables and KAP, we applied the Chi-square test with a conventional significance level of 0.05. However, to avoid over-interpretation of unadjusted associations, only descriptive trends are emphasized in the results and discussion.

## 3 Results

### 3.1 Socio-demographic information of participants

This study involved 516 individuals and healthcare professionals. Young participants made up the majority, with 82.6% aged between 23 and 25 years; participation among older individuals (aged 56 and above) was minimal at just 1.7%. Males comprised a larger portion of the participants at 75.6%, while females accounted for 24.4%. Most respondents held a Bachelor’s degree in Medicine (MBBS) at 61.4%, with fewer holding a Bachelor’s in Dental Surgery (BDS). Additionally, 56% of the participants had limited experience, and 58.1% were physicians. The majority worked in hospitals (70.3%), and slightly more participants came from non-hotspot areas (53.3%). **Table 1** outlines the demographic characteristics of those studied.

**Table 1:** Socio-demographic information of participants (N= 516).

S. No	Variable	Unique Variable	Frequency	Percentage
1	Age (Years)	23-35	389	75.4
		36-45	82	15.9
		46-55	36	7
		56 or above	9	1.7
2	Gender	Female	126	24.4
		Male	390	75.6
3	Qualification	Bachelor (MBBS)	317	61.4
		Bachelor (BDS)	18	3.5
		Master	81	15.7
		Doctorate	100	19.4
4	Years of Experience	<5	289	56
		5-10	182	35.3
		11-15	27	5.2
		>15	18	3.5
5	Profession	Physician	300	58.1
		Public Health Officer	117	22.7
		Other	99	19.2
6	Working Setting/place	Hospital	363	70.3
		Clinic	45	8.7
		Community Health Centre	36	7.0
		Other	72	14.0
7	Zone of Duty	Hot Spot	241	46.7
		Non Hot Spot	275	53.3

### 3.2 Overall score of knowledge, attitude, and perception level

**Table 2** illustrates the overall scores regarding participants’ knowledge, attitudes, and perceptions of dengue. The majority of participants (65.1%) displayed medium knowledge about dengue, followed by 19.6% with high knowledge and 15.3% with low knowledge. Regarding attitudes toward dengue, a positive attitude predominated with 72.5% followed by moderate (24.2%), while only 3.3% exhibited a negative attitude toward the disease. A similar trend is observed in participants’ perceptions of dengue, with most (44.2%) expressing moderate perception, although high perception was the least frequent among the respondents.

**Table 2:** Overall score of knowledge, attitude, and perception level.

Characteristic	Score level	Frequency	Percentage
Knowledge	High	101	19.6
	Medium	336	65.1
	Low	79	15.3
Attitude	Negative	17	3.3
	Moderate	125	24.2
	Positive	374	72.5
Practice	Low	197	38.2
	Moderate	228	44.2
	High	91	17.6

### 3.3 Knowledge related to Dengue

**Table 3** summarizes how each question assesses participants’ knowledge of Dengue. In this study, among the 516 participants, 462 (89.5%) identified the virus as the cause of Dengue fever, while 45 (8.7%) attributed it to protozoa, and only 9 (1.7%) cited bacteria as the causative agent. Of the 516 people surveyed, 336 (65.1%) reported having seen the Dengue fever vector, while the rest had not encountered visuals of it. Among the participants, 87.8% identified the *Aedes* mosquito as a potential Dengue vector, compared to 5.2% for Anopheles mosquitoes and 3.5% for Culex. Out of the total participants, 407 (78.9%) described the Dengue mosquito as “a small dark mosquito with stripes on its legs,” while the remaining 109 (21.1%) referred to it as having “lyre-shaped markings on its thorax.” Regarding breeding sites for Dengue mosquitoes, 52.7% of participants indicated that “stagnant clean water” serves as a breeding ground. Similarly, “flowing clean water” was selected by 26.4% as the second most likely breeding site. In comparison, 17.4% and 3.5% chose “stagnant dirty water” and “flowing dirty water” respectively. Out of 516 participants, 300 (58.1%) believed that Dengue mosquitoes bite predominantly in the morning and evening, while 135 (26.2%) identified evening and night as peak biting times, and 72 (14%) claimed that Dengue mosquitoes could bite at any time. All participants (100%) recognized the transmission of Dengue through an infected mosquito bite. A majority of 399 (77.3%) viewed “fever, headache, and joint pain” as common symptoms of Dengue fever, while 54 (10.5%) thought fever was the only symptom. In terms of diagnostic methods for Dengue, 180 (34.9%) mentioned serology, 45 (8.7%) highlighted PCR, and the majority, 291 (56.4%), acknowledged both methods for diagnosing the disease. When it comes to preventive measures against Dengue, 87.8% suggested that “using insect repellent, wearing long sleeves, removing standing water and using mosquito nets” can help prevent the disease, while 7% recommended “using mosquito repellent” alone as a preventive measure.

**Table 3:** Knowledge related to Dengue.

S. No	Question	Value	Frequency	Percentage
1	What is the causative agent of dengue fever?	Virus	462	89.5
		Bacteria	9	1.7
		Protozoa	45	8.7
2	Have you ever seen a dengue vector?	Yes	336	65.1
		NO	180	34.1
3	What is the primary vector for dengue transmission?	*Aedes* mosquito	453	87.8
		*Anopheles* mosquito	27	5.2
		Culex	18	3.5
		Don’t know	18	3.5
4	How does a dengue mosquito look?	Marking in the form of a lyre on its thorax.	109	21.1
		Small dark mosquito with stripes on its legs	407	78.9
5	Where does the dengue mosquito breed?	Flowing clean water	136	26.4
		Stagnant clean water	272	52.7
		Stagnant dirty water	90	17.4
		Flowing dirty water	18	3.5
6	What are the peak biting hours of the *Aedes* mosquito?	Morning and Evening	300	58.1
		Evening and night	135	26.2
		All Day	72	14
		Don’t Know	9	1.7
7	How is dengue transmitted?	With an infected mosquito bite	516	100
8	What are the common symptoms of dengue fever?	Fever, Headache, Joint Pain	399	77.3
		Fever, Headache, Rash	27	5.2
		Fever	54	10.5
		Joint Pain	27	5.2
		Don’t Know	9	1.7
9	Which diagnostic methods are used for dengue detection?	Both	291	56.4
		Serology	180	34.9
		PCR	45	8.7
10	What are preventive measures for dengue?	Using insect repellent, wearing long sleeves, removing standing water, using mosquito nets	453	87.8
		Removing standing water	18	3.5
		Using mosquito nets	9	1.7
		Using mosquito repellent	36	7.0

### 3.4 Attitude toward Dengue

**Table 4** summarizes how each question gauges participants’ attitudes toward Dengue. Data indicates that most participants (51%) perceive Dengue as a moderately serious threat in their area, with 43.8% rating it as a very serious concern, while only 5.2% view it as a non-serious threat. A majority of participants (47.3%) strongly agreed that healthcare professionals are primarily responsible for dengue prevention; 38.5% agreed, 10.7% were neutral, and 3.7% disagreed. Additionally, 48.9% of healthcare professionals reported inadequate implementation of dengue preventive measures, whereas 45.9% reported adequate measures in their facilities, and 5.2% were unaware of the preventive measures in place. Furthermore, 86% of participants emphasized the vital role of public health in controlling dengue, and 80.8% were well acquainted with the WHO clinical management guidelines for dengue. Likewise, 88.1% of participants found these WHO guidelines very beneficial for managing dengue fever.

**Table 4:** Attitude toward Dengue.

S. No	Question	Value	Frequency	Percentage
1	How serious are you considering the threat of dengue in your region?	Moderately serious	263	43.8
		Very serious	226	51.0
		Not serious	27	5.2
2	Do you believe that dengue prevention is primarily the responsibility of healthcare workers)?	Neutral	55	10.7
		Strongly agree	244	47.3
		Agree	198	38.5
		Disagree	19	3.7
3	Do you think that adequate dengue prevention measures are being implemented in your healthcare facility?	Yes	237	45.9
		No	252	48.9
		Don’t know	27	5.2
4	How important is public education in controlling dengue?	Very important	444	86.0
		Important	72	14
5	Are you familiar with the WHO clinical management guidelines for dengue?	Yes	417	80.8
		No	99	19.2
6	Do the WHO guidelines help in managing dengue fever?	Yes	4548	88.1
		No	571	11.1

### 3.5 Perception and practices towards Dengue

**Table 5** outlines each question’s role in assessing participants’ perceptions of Dengue. From a total of 516 participants, 417 (80.8%) consistently educated patients and community members on dengue prevention. In contrast, 8.5% rarely engaged in such education, while 10.5% never did. Notably, 79.1% of respondents always recommended CBC lab tests to monitor patients suspected or confirmed of having dengue, whereas 15.7% did so rarely, and 5.2% never recommended these investigations. A majority (57.2%) reported consistent use of protective measures, such as repellents and nets, at home or work to avoid mosquito bites, while 41.1% stated they used these measures rarely, and only 1.7% indicated they had never used them. Among all participants, a majority claimed their respective facilities performed regular mosquito control activities, although 42.8% noted that such activities were not conducted at their facilities. Regarding actions taken upon suspecting a dengue case, the most common response (38.4%) was a combination of immediate testing (PCR/serology), symptomatic treatment, reporting to public health authorities, and isolating the patient. This was followed by 22.7% who chose immediate testing, symptomatic treatment, and patient isolation, while 21.5% opted for immediate testing alone, and 17.4% reported to Public Health Authorities. Out of 516 respondents, 262 (50.8%) indicated their facilities had sufficient resources for dengue treatment, whereas 191 (37%) disagreed about the presence of such resources. Additionally, among the 516 respondents, 308 had never received formal training in dengue management and prevention, whereas 208 had received training, of which 76.4% had done so occasionally and only 23.6% regularly.

**Table 5:** Perception towards Dengue.

S. No	Question	Value	Frequency	Percentage
1	Do you regularly educate patients and community people about dengue prevention measures?	Always	417	80.8
		Never	54	10.5
		Rarely	45	8.7
2	How frequently do you prefer CBC lab investigation to monitor patients with suspected or confirmed dengue?	Always	408	79.1
		Never	27	5.2
		Rarely	81	15.7
3	Do you regularly educate patients and community people about dengue prevention measures?	Always	314	60.9
		Never	21	4.1
		Rarely	181	35.1
4	How often do you use protective measures (e.g., repellents, and nets) at home or at work to prevent mosquito bites?	Always	295	57.2
		Never	9	1.7
		Rarely	212	41.1
5	Does your healthcare facility conduct regular mosquito control activities (e.g., spraying and removing stagnant water)?	Yes	250	48.4
		No	221	42.8
		Don’t Know	45	8.7
6	What action do you take when diagnosing a suspected dengue case?	Immediate testing (PCR/serology), Symptomatic treatment, Isolation of patient	117	22.7
		Immediate testing (PCR/serology), Symptomatic treatment, Reporting to public health authorities, Isolation of patient	198	38.4
		Immediate testing (PCR/serology)	111	21.5
		Reporting to Public Health Authorities	90	17.4
7	Do you think your facility has sufficient resources (medication, testing kits, etc.) for dengue treatment?	Yes	262	50.8
		No	191	37.0
		Not Sure	63	12.2
8	Have you received any formal training on dengue management and prevention?	Yes	208	40.3
		No	308	59.7
9	How frequently do you attend dengue-related training or workshops?	Occasionally	159	76.4
		Regularly	49	23.6

### 3.6 Association of knowledge, attitude, and perception with the demographic variables

This study examined the relationships among knowledge, attitude, and perception about demographic factors such as age, gender, educational qualifications, years of experience, occupation, workplace, and duty zone. The detailed outcomes of the regression analyses, including demographic factors, are presented in **Supplementary Tables 1**–**3**. **Supplementary Table 1** found a statistically significant association between knowledge and demographic variables. Younger individuals displayed higher knowledge levels regarding Dengue, while older participants showed moderate knowledge, and those aged 56 and older had limited representation, with only 1.7% indicating medium knowledge and none indicating high knowledge. Gender differences were significant, with males exhibiting higher levels of both medium and high knowledge than females. Educational qualifications and experience were also crucial, as higher, and medium knowledge levels were most common among participants with bachelor’s degrees (MBBS). Moreover, those with less than five years of experience showed a higher percentage of medium knowledge, while individuals with five to ten years of experience were more likely to demonstrate high knowledge. Physicians showed higher and moderate knowledge levels about dengue, followed by Public Health Officers. Higher and moderate knowledge levels were notably prevalent among participants working in hospitals, followed by those in community health centers and clinics, while health professionals in Non-Hot spot areas exhibited more knowledge than those in Hot-Spot zones.

**Supplementary Table 2** revealed a significant association between attitudes and demographic factors. Younger participants expressed more positive attitudes towards dengue fever, while older participants showed moderate attitudes. Similarly, knowledge and positive attitudes were more common among male participants compared to females. Participants with MBBS qualifications and under five years of experience also displayed positive attitudes. Additionally, physicians and public health officers demonstrated favorable attitudes toward dengue fever, as did participants working in hospitals and those stationed in Hot-Spot zones.

**Supplementary Table 3** indicated a significant association between practice/perception and demographic variables. Knowledge, attitude, and positive perception were noted among younger and male participants, while those with MBBS qualifications and 5 to 10 years of experience also exhibited positive perceptions. Furthermore, physicians, hospital-based workers, and those in Hot-Spot zones also showed positive perceptions.

## 4 Discussion

Over recent decades, the rapid rise in dengue cases worldwide has elevated it to a significant global public health concern. Recent research was conducted in Khyber Pakhtoonkhwa, Pakistan, to assess health professionals’ knowledge, attitudes, and behaviors towards dengue, a prevalent viral infection in humans transmitted by mosquitoes and a major public health issue. In this cross-sectional study, male participants outnumbered females, consistent with Ali et al. (2024), who reported that 76% of participants were male (Ali et al., 2024). Our research similarly matches the findings from a study in the Malakand region of Pakistan, where male participants were the majority (Zohra et al., 2024). Nevertheless, our research study varied somewhat from the KAP-based investigation on dengue fever conducted among the local population of Karachi, Pakistan (Ali et al., 2023). Unlike our research, a study conducted in Nepal found that female participation was higher, at 64.2%, compared to male participation (Phuyal et al., 2022). A study conducted in Shabwah Governorate, Yemen, also noted significant participation by women regarding the community’s knowledge, attitudes, and practices toward dengue fever (Saghir et al., 2022). Our current study indicates that a significant proportion of participants demonstrated high knowledge (19.6%) and medium knowledge (65.1%) about dengue. Supporting our findings, a similar study observed a high level of dengue knowledge among participants (Zeb et al., 2024). Our research corresponds with findings from Australia, India, and Pakistan, indicating that most people are aware of Dengue (Syed et al., 2010; Jeelani et al., 2015; Gyawali et al., 2016). In agreement with our study, over fifty percent of participants demonstrated strong knowledge of dengue (Saghir et al., 2022). Conversely, a study in Nepal found that just 2.3% of households showed high awareness of dengue, whereas a considerable number had low (37.9%) or no knowledge (59.8%) (Phuyal et al., 2022). A similar study was conducted in eastern Nepal, which shows that a small percentage of participants were aware of dengue (Heera and Parajuli, 2016). A study in Pakistan revealed that many medical students were uninformed about dengue, probably due to insufficient awareness programs (Saghir et al., 2022). Our study’s high level of dengue knowledge is likely because our participants are health professionals. Conversely, the low dengue knowledge observed in other studies could stem from the fact that those countries experienced dengue cases in the early 21st century, whereas countries with higher knowledge encountered cases in the 20th century (Malla et al., 2008). The main carriers of dengue transmission to humans are *Aedes aegypti* and *Aedes albopictus*. In our study, most participants identified the *Aedes* mosquito as the main vector for dengue transmission. This finding aligns with research from Malaysia, where a significant portion of participants (87.8%) recognized the vector linked to dengue. However, in contrast to our results, Saghir et al. (2022) found that only 75% of their respondents attributed dengue transmission to *Aedes* mosquitoes (Saghir et al., 2022). In a different study, a small number of participants recognized *Aedes* mosquitoes as the primary carriers of dengue transmission (Phuyal et al., 2022). Regarding the feeding habits of dengue vectors, these mosquitoes primarily feed in the early morning and late afternoon (Rogozi, 2023). In our research, over half of the participants identified morning and evening as the prime times for mosquito bites. This finding aligns with earlier studies that showed more than 50% of participants were aware of mosquitoes’ biting times (Phuyal et al., 2022). A different study found that 56% of participants know that dengue mosquitoes typically feed or bite during the day (Saghir et al., 2022). A study in rural Cambodia found that 74% of participants thought the dengue vector bites in the daytime (Kumaran et al., 2018). *Aedes* species usually reproduce in various natural and artificial containers that hold clean, stagnant water. Common breeding sites are ant traps, earthen jars, flower pots, drums, concrete tanks, coconut shells, and old tires (Madzlan et al., 2016). Our study revealed that over half of the participants were knowledgeable about the breeding habitats of *Aedes* mosquitoes, accurately identifying “stagnant clean water” as a prevalent breeding site. They understood the significance of removing these habitats as a crucial strategy for dengue prevention. These results are consistent with a study carried out in Nepal between 2011 and 2012 (Dhimal et al., 2014). These results align with earlier studies carried out in Taiz (Alyousefi et al., 2016), Southern Thailand (Suwanbamrung et al., 2013), and highland and lowland communities in Central Nepal (Dhimal et al., 2014). “Stagnant water and keeping water containers open” contribution to dengue spread was also assured (Saghir et al., 2022). Likewise, several studies conducted in Taiz, and rural Cambodia have found knowledge levels regarding dengue preventive measures and diverse breeding habitats (Alyousefi et al., 2016; Kumaran et al., 2018). In terms of preventive measures for dengue, the majority of participants in our study demonstrated knowledge of the correct answers. This finding aligns with Saghir et al. (2022), which also showed that most participants selected the right answers (Saghir et al., 2022). A cross-sectional study was carried out in Pakistan, revealing that participants had a good understanding of dengue fever and were knowledgeable about its prevention methods (Hassan et al., 2021). Most participants recognized additional common signs and symptoms of Dengue fever besides fever and headache, while only a few could identify fever and headache. Supporting our study, a cross-sectional study in Pakistan indicated that their participants were similarly aware of dengue fever signs and symptoms (Naqvi et al., 2024). In contrast, another study found that only a small number of community participants could identify the signs and symptoms of dengue (Phuyal et al., 2022). In our study on diagnostic methods for detecting dengue, most participants chose serology and PCR. However, unlike our findings, a study from Dera Ismail Khan, Pakistan, found that most students could not accurately identify the confirmatory test for dengue detection (Ali et al., 2024).

The positive attitude scores for most participants (72.2%) reflect increased public concern about dengue, aligning with comparable results from Pakistan (Qureshi, 2014) and Yemen (Saied et al., 2015). The majority of participants viewed dengue as a significant health issue and showed considerable backing for prevention and control measures. This study aligns with the findings of which also regard dengue as a serious condition (Abd Rahman et al., 2014). Our study aligns with the findings of Saghir et al. (2022), who noted that most participants had a positive attitude toward dengue (Saghir et al., 2022). Our study mirrored earlier research from Yemen’s Taiz and Hodeidah governorates, highlighting a favorable perception of the seriousness and spread of DF, along with its prevention and community involvement (Saied et al., 2015; Alyousefi et al., 2016).

The majority of participants in our study reported medium to high practice scores related to dengue. This aligns with previous findings that most participants demonstrated a strong awareness of dengue (Saghir et al., 2022). In contrast to earlier studies in Yemen that noted minimal community engagement (Saied et al., 2015; Alyousefi et al., 2016), Our study indicates a substantial level of community practice, likely shaped by local traditions, culture, education, and recent interactions with other governorates. In contrast to our findings, a previous study in Nepal found low practice scores regarding dengue (Phuyal et al., 2022). Likewise, a separate study conducted in Jamaica noted decreased practices regarding dengue prevention. (Shuaib et al., 2010) and Indonesia (Harapan et al., 2018).

In our current study, males demonstrated greater knowledge, more positive attitudes, and improved practices than females. Similarly, in another study, they identified that gender influences behaviors related to dengue prevention, likely stemming from social behaviors and individual habits (Banik et al., 2023). In contrast to our findings, previous research reported that gender did not significantly influence knowledge, attitudes, and practices regarding dengue (Ali et al., 2024). In a similar vein, Alvarado-Castro et al. (2024) noted that males and females share an equal understanding of dengue fever (Alvarado-Castro et al., 2024).

## 5 Study limitations and strength

This study has several notable strengths alongside limitations. On the strength side: 1) The inclusion of 516 participants allows reasonable statistical power. 2) Cronbach’s Alpha analysis confirmed the internal consistency of the survey. 3) Both descriptive and inferential statistics were employed effectively. However, the study also presented some limitations: 1) We used a cross-sectional design rather than a longitudinal approach, which limits the ability to assess changes over time. 2) The survey was distributed via social media, potentially excluding individuals who do not access these platforms.

## 6 Recommendation

Based on the evaluation of the KAP of medical professionals regarding dengue and its vector, we proposed the following recommendations for future research and practice. 1) There should be regular and structural training programs to continually update and enhance professional skills. 2) Standardize and periodically update dengue management protocols to ensure best practices are maintained. 3) Enhanced community education and outreach facility at the district or tehsil level to improve public awareness. 4) Modernize the diagnostic facility at both the central and the hospital levels. 5) Organize a seminar and conferences focused on the significance of the One Health approach. 6) Establishment of One health center to coordinate joint activities, bringing together veterinary, Para veterinary, medical, and paramedical professionals for collaborative initiatives.

## 7 Conclusion

This cross-sectional study examines the knowledge, attitudes, and practices of medical professionals in Khyber Pakhtunkhwa regarding dengue and its prevention. Although most participants exhibited moderate to high knowledge levels and favorable attitudes towards dengue management, noticeable gaps in perceptions and practices were found, especially in consistently implementing protective measures and receiving formal training on dengue prevention and management. Enhancing training programs, optimizing resource distribution, and promoting public health education can improve readiness and effective responses to dengue outbreaks in the area. These results highlight the necessity for integrated One Health strategies to reduce the impact of dengue and other vector-borne diseases.

## Data Availability

The datasets presented in this study can be found in online repositories. The names of the repository/repositories and accession number(s) can be found in the article/**Supplementary Material**.
