# Supplementary material for: Knowledge, attitudes, and practices regarding dengue and its vectors among medical professionals: a cross-sectional study
Source: Front Cell Infect Microbiol. 2025 Apr 30;15:1560054. doi: 10.3389/fcimb.2025.1560054 (PMC12075356; doi:10.3389/fcimb.2025.1560054)
Supplement: Supplementary file 1 [file Table1.docx]

**Knowledge, Attitudes, and Practices regarding Dengue and its vectors among medical professionals: A Cross-Sectional Study**

Rania Ali El Hadi Mohamed^1^, Yasir Khan^2^, Khalid J. Alzahrani^3^, Fuad M. Alzahrani^3^, Khalaf F. Alsharif^3^, Aamir Khan^4^, Fazal Noor^5^, Abdul Qadeer^5*^, Geng-Bai Lin^6*^, Chien-Chin Chen^7,8,9,10*^

1. Department of Biology, College of Science, Princess Nourah bint Abdulrahman University, P.O.Box 84428, Riyadh 11671, Saudi Arabia. Email: (RAEHM: rasaddig@pnu.edu.sa)
2. Lady Reading Hospital Peshawar-Pakistan, Email: (YK: [yawan4526@gmail.com](mailto:yawan4526@gmail.com))
3. Department of Clinical Laboratories Sciences, College of Applied Medical Sciences, Taif University, P.O. Box 11099, Taif 21944, Saudi Arabia. Emails ([Ak.jamaan@tu.edu.sa](mailto:Ak.jamaan@tu.edu.sa), FA: [Fuadmubarak@tu.edu.sa](mailto:Fuadmubarak@tu.edu.sa), KA: [alsharif@tu.edu.sa](mailto:alsharif@tu.edu.sa))
4. Livestock and Dairy Development Department (Extension wing), Khyber Pakhtunkhwa, Pakistan Email: (AK: [aamirkhanbannuzai@gmail.com](mailto:aamirkhanbannuzai@gmail.com) )
5. Livestock and Dairy Development Department (Research wing), Khyber Pakhtunkhwa, Pakistan Email: (FN: [fazalnoor555@gmail.com](mailto:fazalnoor555@gmail.com) )
6. Department of Cell Biology, School of Life Sciences, Central South University, Changsha, The People's Republic of China. Email: (AQ: [qadeerktk848@yahoo.com](mailto:qadeerktk848@yahoo.com) )
7. Division of Surgical Critical Care, Department of Surgery, Ditmanson Medical Foundation Chia-Yi Christian Hospital, Chiayi 600, Taiwan. Email: (GBL: shoubai2006@gmail.com)
8. Department of Pathology, Ditmanson Medical Foundation Chia-Yi Christian Hospital, Chiayi 600, Taiwan; Email: (CCC: [hlmarkc@gmail.com](mailto:hlmarkc@gmail.com))
9. Department of Cosmetic Science, Chia Nan University of Pharmacy and Science, Tainan 717, Taiwan.
10. Doctoral Program in Translational Medicine, National Chung Hsing University, Taichung 402, Taiwan.
11. Department of Biotechnology and Bioindustry Sciences, College of Bioscience and Biotechnology, National Cheng Kung University, Tainan, 701, Taiwan.

Supplementary Material

S. Table 1. Correlation between Knowledge and demographic variables (N=516)

| **S. No** | **Variable** | **Unique Variable** | **Knowledge** | | | | | | **P Value** | **Chi Square** |
| --- | --- | --- | --- | --- | --- | --- | --- | --- | --- | --- |
|  |  |  | **High** | **Medium** | | | **Low** | |  |  |
| 1 | Age (Years) | 23-35 | 64 (12.4%) | 273 (52.9%) | | | 52 (10.1) | | 0.00 | 54.408 |
|  |  | 36-45 | 28 (5.4%) | 27 (5.2%) | | | 27 (5.2%) | |  |  |
|  |  | 46-55 | 9 (1.7%) | 27 (5.2%) | | | 0 (0.00%) | |  |  |
|  |  | 56 or above | 0 (0.00%) | 9 (1.7%) | | | 0 (0.00%) | |  |  |
| 2 | Gender | Female | 45 (8.7%) | 45 (8.7%) | | | 36 (7.0%) | | 0.000 | 63.470 |
|  |  | Male | 56 (10.9%) | 291 (56.4) | | | 43 (8.3%) | |  |  |
| 3 | Qualification | Bachelor (MBBS) | 56 (10.9%) | 227 (44.0%) | | | 34 (6.6%) | | 0.00 | 128.713 |
|  |  | Bachelor (BDS) | 0 (0.00%) | 9 (1.7%) | | | 9 (1.7%) | |  |  |
|  |  | Master | 45 (8.7%) | 18 (3.4%) | | | 18 (3.4%) | |  |  |
|  |  | Doctorate | 18 (3.5%) | 82 (15.9%) | | | 0 (0.00%) | |  |  |
| 4 | Years of Experience | <5 | 36 (7.0%) | | 201 (39.0%) | | 52(10.1%) | | 0.000 | 41.002 |
|  |  | 5-10 | 56 (10.9%) | | 99(19.2%) | | 27(5.2%) | |  |  |
|  |  | 11-15 | 9 (1.7%) | | 18 (3.5%) | | 0 (0.00%) | |  |  |
|  |  | >15 | 0 (0.00%) | | 18 (3.5%) | | 0 (0.00%) | |  |  |
| 5 | Profession | Physician | 47 (9.1%) | | | 235 (45.5%) | | 18 (3.5%) | 0.00 | 96.167 |
|  |  | Public Health Officer | 45 (8.7%) | | | 45 (8.7%) | | 27 (5.2%) |  |  |
|  |  | Other | 9 (1.7%) | | | 56 (10.9%) | | 34 (6.6%) |  |  |
| 6 | Working Setting | Hospital | 56 (10.9%) | | | 271 (52.5%) | | 36 (7.0%) | 0.000 | 161.444 |
|  |  | Clinic | 0 (0.00%) | | | 36 (7.0%) | | 9 (1.7%) |  |  |
|  |  | Community Health Centre | 27 (5.2%) | | | 9 (1.7%) | | 0 (0.00%) |  |  |
|  |  | Other | 18 (3.5%) | | | 20 (3.9%) | | 34 (6.6%) |  |  |
| 7 | Zone of Duty | Hot Spot | 36 (7.0%) | | | 178 (34.5%) | | 27 (5.2%) | 0.000 | 15.255 |
|  |  | Non Hot Spot | 65 (12.6%) | | | 158 (30.6%) | | 52 (10.1%) |  |  |

S. Table 2. Correlation between Attitude and demographic variables (N=516)

| **S. No** | **Variable** | **Unique Variable** | **Attitude** | | | **P Value** | **Chi Square** |
| --- | --- | --- | --- | --- | --- | --- | --- |
|  |  |  | **Negative** | **Moderate** | **Positive** |  |  |
| 1 | Age (Years) | 23-35 | 8 (1.6%) | 116 (22.5%) | 256 (51.4) | 0.00 | 45.919 |
|  |  | 36-45 | 9 (1.7%) | 9 (1.7%) | 64 (12.4%) |  |  |
|  |  | 46-55 | 0 (0.00%) | 0 (0.00%) | 36 (7.0%) |  |  |
|  |  | 56 or above | 0 (0.00%) | 0 (0.00%) | 9 (1.7%) |  |  |
| 2 | Gender | Female | 9 (1.7%) | 28 (5.4%) | 89 (17.2%) | 0.020 | 7.848 |
|  |  | Male | 8 (1.6%) | 97 (18.8) | 285 (55.2%) |  |  |
| 3 | Qualification | Bachelor (MBBS) | 8 (1.6%) | 86 (16.7%) | 223 (43.2%) | 0.00 | 139.186 |
|  |  | Bachelor (BDS) | 9 (1.7%) | 0 (0.00%) | 9 (1.7%) |  |  |
|  |  | Master | 0 (0.00%) | 11 (2.1%) | 70 (13.6%) |  |  |
|  |  | Doctorate | 0 (0.00%) | 28 (5.4%) | 72 (14.0%) |  |  |
| 4 | Years of Experience | <5 | 8 (1.6%) | 105 (20.3%) | 176(34.1%) | 0.000 | 58.201 |
|  |  | 5-10 | 9 (1.7%) | 20 (3.9%) | 153 (29.7%) |  |  |
|  |  | 11-15 | 0 (0.00%) | 0 (0.00%) | 27 (5.2%) |  |  |
|  |  | >15 | 0 (0.00%) | 0 (0.00%) | 18 (3.5%) |  |  |
| 5 | Profession | Physician | 17 (3.3%) | 60 (11.6%) | 223 (43.5%) | 0.001 | 18.190 |
|  |  | Public Health Officer | 0 (0.00%) | 37 (7.2%) | 80 (15.5%) |  |  |
|  |  | Other | 0 (0.00%) | 28 (5.4 %) | 71 (13.8%) |  |  |
| 6 | Working Setting | Hospital | 8 (1.6%) | 78 (15.1%) | 277(53.7%) | 0.000 | 89.193 |
|  |  | Clinic | 9 (1.7%) | 9 (1.7%) | 27 (5.2%) |  |  |
|  |  | Community Health Centre | 0 (0.00%) | 0 (0.00%) | 36 (7.0%) |  |  |
|  |  | Other | 0 (0.00%) | 38 (7.4%) | 34 (6.6%) |  |  |
| 7 | Zone of Duty | Hot Spot | 9 (1.7%) | 75 (14.5%) | 157 (30.4%) | 0.02 | 12.498 |
|  |  | Non Hot Spot | 8 (1.6%) | 50 (9.7%) | 217 (42.1%) |  |  |

S. Table 3. Correlation between Practices and demographic variables (N=516)

| **S. No** | **Variable** | **Unique Variable** | **Practice** | | | **P Value** | **Chi Square** |
| --- | --- | --- | --- | --- | --- | --- | --- |
|  |  |  | **Low** | **Medium** | **High** |  |  |
| 1 | Age (Years) | 23-35 | 154 (29.8%) | 172 (33.3%) | 63 (12.2) | 0.002 | 21.394 |
|  |  | 36-45 | 25 (4.8%) | 39 (7.6%) | 18 (3.5%) |  |  |
|  |  | 46-55 | 9 (1.7%) | 17 (3.3%) | 10 (1.9%) |  |  |
|  |  | 56 or above | 9 (1.7%) | 0 (0.00%) | 0 (0.00%) |  |  |
| 2 | Gender | Female | 31 (6.0%) | 67 (13.0%) | 28 (5.4%) | 0.001 | 13.084 |
|  |  | Male | 116 (32.2%) | 161 (31.2) | 63 (12.2%) |  |  |
| 3 | Qualification | Bachelor (MBBS) | 101 (19.6%) | 152 (29.5.%) | 64 (12.4%) | 0.00 | 33.689 |
|  |  | Bachelor (BDS) | 9 (1.7%) | 9 (1.7%) | 0 (0.00%) |  |  |
|  |  | Master | 26 (5.0%) | 37 (7.2%) | 18 (3.5%) |  |  |
|  |  | Doctorate | 61 (11.8%) | 30 (5.8%) | 9 (1.7%) |  |  |
| 4 | Years of Experience | <5 | 119 (23.1%) | 145 (28.1%) | 25 (4.8%) | 0.000 | 62.531 |
|  |  | 5-10 | 60 (11.6%) | 66 (12.8%) | 56 (10.9%) |  |  |
|  |  | 11-15 | 9 (1.7%) | 17 (3.3%) | 1 (0.2%) |  |  |
|  |  | >15 | 9 (1.7%) | 0 (0.00%) | 9 (1.7%) |  |  |
| 5 | Profession | Physician | 96 (18.6%) | 153 (29.7%) | 51 (9.9%) | 0.00 | 21.306 |
|  |  | Public Health Officer | 48 (9.3%) | 41 (7.9%) | 28 (5.4%) |  |  |
|  |  | Other | 53 (10.3%) | 34 (6.6%) | 12 (2.3%) |  |  |
| 6 | Working Setting | Hospital | 113 (21.9%) | 1911 (37.0%) | 59 (11.4%) | 0.000 | 84.801 |
|  |  | Clinic | 34 (6.6%) | 10 (1.9%) | 1 (0.2%) |  |  |
|  |  | Community Health Centre | 7 (1.4%) | 10 (1.9%) | 19 (3.7%) |  |  |
|  |  | Other | 43 (8.3%) | 17 (3.3%) | 12 (2.3%) |  |  |
| 7 | Zone of Duty | Hot Spot | 84 (16.3%) | 103 (20.0%) | 54 (10.5%) | 0.025 | 7.359 |
|  |  | Non Hot Spot | 113 (21.9%) | 125 (24.2.6%) | 37 (7.2%) |  |  |
